# Higher Levels of High-Sensitivity C-Reactive Protein Is Positively Associated with the Incidence of Hyperuricemia in Chinese Population: A Report from the China Health and Retirement Longitudinal Study

**DOI:** 10.1155/2020/3854982

**Published:** 2020-05-20

**Authors:** Hui-Xu Dai, Zhi-Ying Zhao, Yang Xia, Qi-Jun Wu, Yu-Hong Zhao

**Affiliations:** ^1^Department of Clinical Epidemiology, Shengjing Hospital of China Medical University, Shenyang, China; ^2^Clinical Research Center, Shengjing Hospital of China Medical University, Shenyang, China

## Abstract

**Purpose:**

The aim of the present cohort study was to explore the longitudinal association between high-sensitivity C-reactive protein (CRP) and hyperuricemia in Chinese population. Furthermore, we conducted subgroup analyses to explore this association according to age, sex, and body mass index.

**Methods:**

A total of 5,419 healthy participants were enrolled in the final cohort analysis. The high-sensitivity CRP level was measured by immunoturbidimetric assay. Hyperuricemia was defined as serum uric acid ≥7.0 mg/dL (416 *μ*mol/L) in men and ≥6.0 mg/dL (357 *μ*mol/L) in women. Multivariate logistic regression was used to analyze the association.

**Results:**

During the 4 years follow-up, 474 participants developed hyperuricemia. Compared with participants in the lowest tertile of high-sensitivity CRP, the multivariate-adjusted odds ratio (OR) (95% confidence interval [CI]) for incident hyperuricemia in the highest tertile was 1.36 (1.02, 1.82). In the subgroup analyses, high-sensitivity CRP was positively associated with the incidence of hyperuricemia after multivariate adjustments (*P* for trend = 0.04) in women. Compared with the women in the lowest tertile of high-sensitivity CRP, the multivariate-adjusted OR (95% CI) in the highest tertile was 1.69 (1.10, 2.66). No statistically significant association was found in other subgroups.

**Conclusions:**

The findings of this prospective cohort study suggest that higher level of high-sensitivity CRP is an independent risk factor for hyperuricemia in Chinese, especially in women.

## 1. Introduction

Hyperuricemia, as an important chronic disease, threatens human health due to its association with increased risks of multiple comorbidities and mortality [1, 2]. Substantial evidence suggests that hyperuricemia not only plays an important role in the development of gout but is also significantly associated with metabolic syndrome [3–5], chronic kidney disease [6–8], hypertension [9, 10], and cardiovascular diseases [11–14]. The reported prevalence of hyperuricemia in different countries varies from 2.6% to 36.0% and has increased rapidly worldwide in the past few decades [15, 16]. Therefore, the prevention of hyperuricemia is of significance to public health.

High-sensitivity C-reactive protein (CRP), as a general marker of both acute and low-grade chronic inflammation [17], its relationship to hyperuricemia may be bidirectional. Several previous studies [18–22] had shown that CRP was positively associated with the prevalence of hyperuricemia or the high level of uric acid. While another study [23] has suggested that increased uric acid was not correlated with CRP. However, the design of previous studies has been limited in determining a causal association between high-sensitivity CRP and hyperuricemia. To the best of our knowledge, there were only few cohort studies [24, 25] focus on this association. One prospective study [25] included 4154 participants aged ≥55 years from the Rotterdam Study suggested that increased levels of uric acid were associated with higher levels of high-sensitivity CRP. However, as the most reliable marker for inflammation in the clinical practice [22], whether CRP is a cause of risk but not merely a symptom for hyperuricemia has an important preventive significance. Another cohort study found that high-sensitivity CRP may serve as a candidate risk predictor for hyperuricemia in middle-aged South Korean men [24], while the association in the older and females has not been reported. However, several studies [22, 26] have shown that CRP differ in age and sex. Therefore, the aim of the present cohort study was to explore the longitudinal association between high-sensitivity CRP and hyperuricemia in Chinese population. Furthermore, we conducted the subgroup analyses to explore this association according to age, sex, and body mass index (BMI).

## 2. Materials and Methods

### 2.1. Source of Data

This cohort study used data from the China Health and Retirement Longitudinal Study (CHARLS) [27], which was a longitudinal survey in China. The national baseline survey for CHARLS was conducted between June 2011 and March 2012, and 17,708 participants aged 45 years and older were involved. The participants were selected using a multistage probability sampling method from 450 counties of 28 provinces, and the response rate was 80.5%. Participants were followed every two years, and blood sample collection was done once in every two follow-up periods (4 years). Details of CHARLS were published previously [27].

### 2.2. Study Population

**Figure 1:**
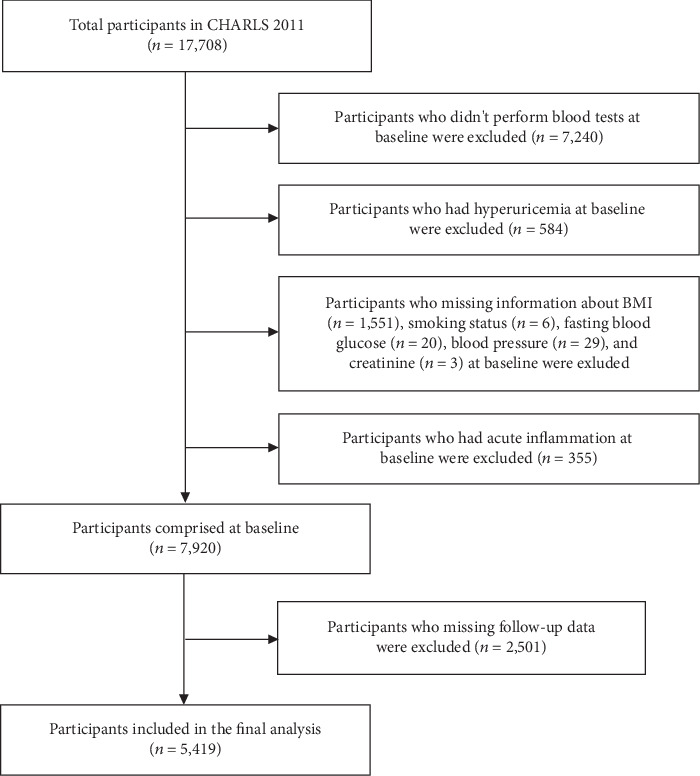
Flow chart of the selection of study participants. CHARLS, China Health and Retirement Longitudinal Study; BMI, body mass index.

CHARLS included 17,708 participants, and a total of 10,468 blood tests that had been performed at baseline. For this study, we excluded participants who did not provide information on BMI (*n* = 1,551), smoking status (*n* = 6), fasting blood glucose (*n* = 20), blood pressure (*n* = 29), creatinine (*n* = 3). Besides, participants who had hyperuricemia (*n* = 584) or those who had acute inflammation (high-sensitivity CRP level >10.0 mg/L) [28] (*n* = 355) at baseline were also excluded. Thus, the cohort comprised 7,920 participants at baseline. Furthermore, 2,501 (31.6%) participants were excluded because of missing follow-up data. Therefore, 5,419 participants were included in the final cohort analyses (Figure 1). The study protocol was approved by the ethics committee of Peking University, and the participants provided written informed consent before participating in the present study.

### 2.3. Data Collection

The demographic variables and health status functioning in the present study were collected by standardized questionnaires and included sex, age, education level (no formal education, elementary or below, or middle school and above), smoking status (nonsmoker, exsmoker, or current smoker), drinking status (drinking more than once a month, drinking less than once a month, or nondrinker), income (more than 2,433.07 yuan/year or not), marital status (married or unmarried), and living area (urban or rural). Education level was divided into three categories: no formal education, elementary or below, and middle school or above. Income was classified as “≥ mean value” or not and 2433.07 yuan per year was the mean value. Marital status was divided into married and unmarried. Among them, married included “married with spouse present” and “married but not living with spouse temporarily for reasons such as work.” Unmarried included separated, divorced, widowed, and never married. BMI was calculated as weight in kilogram divided by height in meters squared (kg/m^2^). Blood pressure was tested three times, and then the mean value was taken. Hypertension was defined as systolic blood pressure ≥140 mm Hg or diastolic blood pressure ≥90 mm Hg [29]. Fasting plasma glucose was measured by an enzymatic colorimetric test and it was divided into three categories: <110 mg/dL, 110–126 mg/dL, and ≥126 mg/dL [30]. Total cholesterol, triglycerides, and high-density lipoprotein (HDL) cholesterol were all performed by enzymatic colormetric test, and the unit was mg/dL, respectively. Estimate glomerular filtration rate (eGFR) was calculated using the “Xiangya equation” [31].

### 2.4. Assessment of Hyperuricemia

Blood samples were collected from each participant by medically-trained staff from the Chinese Center for Disease Control and Prevention (Chinese CDC); participants had been asked to fast overnight. Venous blood was separated into plasma and buffy coat, immediately stored frozen at -20°C, and transported to the Chinese CDC in Beijing within two weeks where they were stored at -80°C until assayed at Capital Medical University laboratory. Serum uric acid was measured by the UA plus method [27]. Hyperuricemia was defined as serum uric acid ≥7.0 mg/dL (416 *μ*mol/L) in men and ≥6.0 mg/dL (357 *μ*mol/L) in women [32].

### 2.5. Assessment of High-Sensitivity CRP

High-sensitivity CRP was measured by immunoturbidimetric assay [27] in mg/L and was categorized into three tertiles: 0.42 (0.41, 0.43), 0.96 (0.95, 0.97), and 3.13 (3.05, 3.22). According to the tertiles of gender-specific distribution, high-sensitivity CRP level was categorized into 0.44 (0.43, 0.44), 1.00 (0.99, 1.02), and 3.29 (3.16, 3.42) in men and 0.41 (0.40, 0.42), 0.92 (0.91, 0.94), and 3.01 (2.90, 3.12) in women.

### 2.6. Statistical Analyses

The mean and 95% CI were used to describe continuous variables; frequency and percentage were used to describe the categorical variables. Analysis of variance (ANOVA) and chi-squared tests were used to compare the differences between groups at baseline. Multivariate logistic regression was used to analyze the association between high-sensitivity CRP and hyperuricemia. The crude model was used to calculate the crude ORs (95% CIs) without any adjustment. Model 1 was adjusted for age, sex, and BMI. Model 2 was additionally adjusted for education level, smoking status, drinking status, level of income, marital status, fasting blood glucose levels, living area, baseline uric acid, hypertension, total cholesterol, triglycerides, HDL cholesterol, and eGFR based on Model 1. Cubic spline regression model was used to explore the association between high-sensitivity CRP and hyperuricemia when high-sensitivity CRP was taken into account as a continuous exposure [33]. Receiver operating characteristics (ROC) curve was performed to quantify area under the curve (AUC) and optimal cut-off value of high-sensitivity CRP associated with the incidence of hyperuricemia. Multiple linear regression was used to explore the associations between high-sensitivity CRP and the change in uric acid concentrations. Categories of high-sensitivity CRP (coded as “1”, “2”, and “3”) was the independent variable. The change value of uric acid concentrations was the dependent variable. The association between high-sensitivity CRP and hyperuricemia in subgroups according to sex, age, and BMI were further analyzed. 60 years old was used as the cut-off value to conduct the subgroup analyses according to age. The subgroups of BMI was defined according to the Working Group on Obesity in China (normal BMI, <24; high BMI, 24–28; and obesity, ≥28) [34]. All of the statistical analyses were conducted using SAS 9.3 edition (SAS Institute Inc., Cary, NC, USA). All reported *P* values are two sided, and those less than 0.05 were considered significant.

## 3. Results

### 3.1. Participants' Characteristics

A total of 5,419 participants (2,406 males) were included in this study. During the four-year follow-up, 474 participants (8.75%) developed hyperuricemia and the incidence rate was 21.87 per 1,000 person-years. Participants were divided into three groups according to the tertiles of high-sensitivity CRP. Compared with the participants in the lowest tertile of CRP, those in the highest tertile were more likely to be male (*P* for trend <0.01), older (*P* for trend <0.0001), and exsmokers (*P* for trend <0.0001); they also tended to live in urban areas (*P* for trend <0.0001), suffered from hypertension (*P* for trend <0.0001), have higher levels of BMI (*P* for trend <0.0001), fasting plasma glucose (*P* for trend <0.0001), total cholesterol (*P* for trend <0.0001), triglycerides (*P* for trend <0.0001), and uric acid (*P* for trend <0.0001) but lower levels of HDL cholesterol (*P* for trend <0.0001) and eGFR (*P* for trend <0.0001) at baseline (Table 1).

### 3.2. Association between High-Sensitivity CRP and Hyperuricemia Risk

**Figure 2:**
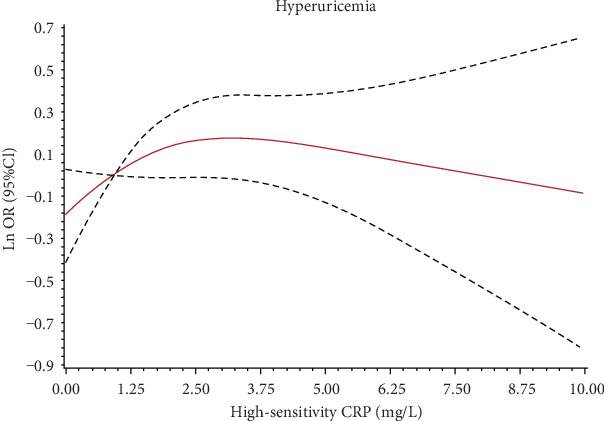
Adjusted association between high-sensitivity C-reactive protein (mg/L) and hyperuricemia. The solid line is ln (OR) and dashed lines are 95 percent confidence intervals.

The association between high-sensitivity CRP and hyperuricemia was shown in Table 2. As shown, the multivariate-adjusted ORs (95% CIs) in model 2 for hyperuricemia incidence across tertiles of high-sensitivity CRP were 1.00 (reference), 1.25 (0.94, 1.68), and 1.36 (1.02, 1.82), respectively. The association between high-sensitivity CRP, which presented as a continuous variable, and hyperuricemia was shown in Figure 2. The overall *P* value and *P* nonlinear were 0.19 and 0.12, respectively.

The optimal cut-off value of high-sensitivity CRP and AUC value for the detection of hyperuricemia were presented in the supplements (Figure S1). As shown, the optimal cut-off value was 0.68. The AUC (95% CI) values was 0.60 (0.58, 0.63).

### 3.3. Association between High-Sensitivity CRP and Change in Uric Acid Concentrations

The association between high-sensitivity CRP and change in uric acid concentrations was shown in Table 3. The results suggested that uric acid concentrations increased in all tertiles of high-sensitivity CRP during the study period. However, the growth rates of uric acid increased across tertiles of high-sensitivity CRP (*P* for trend <0.01). The association between high-sensitivity CRP and change in uric acid concentrations was further explored using multiple linear regression. The high-sensitivity CRP was positively associated with the uric acid change from 2011 to 2015 (*β* = 0.044, standard error = 0.017, *P* < 0.01) after adjustments of confounding factors.

### 3.4. Association between High-Sensitivity CRP and Hyperuricemia Risk according to Sex, Age and BMI

General characteristics of participants in different subgroup analyses according to sex, age, and BMI were supplied in the supplements (Table S1-S3). The association between high-sensitivity CRP and hyperuricemia risk according to sex, age, and BMI were shown in Table 4. High-sensitivity CRP was positively associated with the incidence of hyperuricemia after multivariate adjustments (*P* for trend = 0.04) in women. Compared with the women in the lowest tertile of high-sensitivity CRP, the multivariate-adjusted ORs (95% CIs) in the highest tertile was 1.69 (1.10, 2.66). No statistically significant association was found in other subgroups and all values of *P*-interaction were nonsignificant.

## 4. Discussion

To the best of our knowledge, the present study is the first cohort study to explore the longitudinal association between higher levels of high-sensitivity CRP and hyperuricemia in Chinese. In the present study, we suggested that higher level of high-sensitivity CRP is an independent risk factor for hyperuricemia in Chinese women, while it is a limited indicator for prediction.

The nonsignificant association between high-sensitivity CRP and the incidence of hyperuricemia may attribute to the participants who had acute inflammation (high-sensitivity CRP level >10.0 mg/L) were excluded at baseline, which means all the participants included in the present study had little difference in inflammation levels. However, hyperuricemia in the highest tertile group of high-sensitivity CRP was statistically significant compared with the lowest tertile group. As can be seen from Table 1, the highest tertile group of high-sensitivity CRP was obviously higher than that in the lowest group, indicating that the participants in the highest tertile of CRP were in an intercritical state. In other words, although all we included in the present study were participants with little difference in inflammation levels, the participants in the highest tertile were in a higher level of inflammation. Besides, based on 5,419 participants in the baseline of this study, there was only 474 participants (8.75%) developed hyperuricemia. The lower incidence of hyperuricemia may contribute to the nonsignificant *P* values in the cubic spline regression model. In addition, hyperuricemia is a complex disease with numerous causes, while inflammation is only one aspect of that. Therefore, although CRP is a risk factor for hyperuricemia, its ability to predict hyperuricemia is limited.

Consistent with previous cross-sectional studies [18, 19, 35], we found that higher level of high-sensitivity CRP is an independent risk factor for hyperuricemia. For example, one cross-sectional study of 1935 subjects suggested that high-sensitivity CRP is positively associated with the prevalence of hyperuricemia. In that study, the relative odds of the prevalence of hyperuricemia increased 0.56, 0.55, and 0.96 times in the third, fourth, and fifth quintile, respectively [18]. Another study [19] reached a similar conclusion as the hyperuricemia patients had higher levels of CRP than the controls. However, given the study's cross-sectional design, it was limited in concluding a causal correlation. To the best of our knowledge, there was only one cohort study [24] has shown that elevated high-sensitivity CRP is an independent risk factor for hyperuricemia. The multivariable-adjusted hazard ratio comparing the highest quartiles in the distribution with the lowest was 1.14 (95%CI = 1.02–1.28, *P* for trend = 0.07) for high-sensitivity CRP, which was similar to our results. However, that study merely focused on the middle-aged males in Korea, the older and the female had not been conducted yet. To explore whether there were interaction effects between sex, age, BMI, and high-sensitivity CRP on hyperuricemia, we performed subgroup analyses. In the subgroup analyses of the present study, we found that high-sensitivity CRP was positively associated with the incidence of hyperuricemia after multivariate adjustments (*P* for trend = 0.04) in women. Besides, compared with the women in the lowest tertile of high-sensitivity CRP, the multivariate-adjusted ORs (95% CIs) in the highest tertile were 1.69 (1.10, 2.66). However, the interaction effects between sex, age, BMI, and high-sensitivity CRP on hyperuricemia were nonsignificant. Moreover, almost all the adjusted ORs were not statistically significant (except for the highest tertile in women), which may attribute to the small sample size after stratification and result in low statistical power. Further cohort studies with enough sample size are needed to confirm our results.

The mechanisms that have been proposed to explain these results may related to inflammation. It is generally believed that persistent low-grade inflammation plays an important role in the formation of hyperuricemia [36]. One study [37] has shown that inflammatory cytokines may improve the activity by upregulating the gene expression of xanthine oxidase, which plays a key role in increasing uric acid production. Another study [38] has suggested that xanthine oxidase activity is positively correlated with CRP. In the present study, the mean age of women was 57.23 (56.91, 57.55) years, which means most of the female participants are in postmenopause. Previous study [39] had shown that estrogen has anti-inflammatory effects, and thus, postmenopausal women have elevated inflammation maybe due to low estrogen levels [40]. However, the exact mechanism of the role of CRP in hyperuricemia and the differences between gender are still unclear.

The present study has several strengths. Above all, it is the first study to examine the association between high-sensitivity CRP and the incidence of hyperuricemia by regarding high-sensitivity CRP as the primary exposure in Chinese population. Second, the large sample size in the present study (5,419 participants) allowed for sufficient statistical power to detect associations between high-sensitivity CRP and hyperuricemia. Thirdly, we further analyzed the comprehensive associations between high-sensitivity CRP and hyperuricemia in different subgroups according to sex, age, and BMI. Several limitations should also be acknowledged. First, no information on diet was available; therefore, the potential impact of diet on the results cannot be excluded. Dietary factors include seafood, red meat [41], and fructose, which influence uric acid levels by accelerating the catabolism of adenine nucleotides [42]. Second, there is no information on the use of uric acid-lowering drugs or treatments in the database, so we cannot analyze the effect of this factor on the results. Third, during the follow-up, some factors, such as the change of lifestyle and the treatment of hyperuricemia, may confound the association. Fourth, 32.1% of participants (from 8,307 to 5,643) were excluded due to the lack of follow-up data. Participants who were missing during follow-up could have had different characteristics to those eventually included, and these differences could have impacted the results of this study.

## 5. Conclusions

The findings of this prospective cohort study suggested that higher level of high-sensitivity CRP is an independent risk factor for hyperuricemia in Chinese population, especially in women, while it is a limited indicator for prediction.

## Figures and Tables

**Table 1:** General characteristics of participants according to tertiles of high-sensitivity CRP^a^.

	Tertiles of high-sensitivity CRP (mg/L)			*P* trend^b^
	Tertile 1 (*n* = 1800)	Tertile 2 (*n* = 1810)	Tertile 3 (*n* = 1809)	
High-sensitivity CRP (mg/L)	0.42 (0.41, 0.43)^c^	0.96 (0.95, 0.97)	3.13 (3.05, 3.22)	
Male, *n* (%)	757 (42.06%)	793 (43.81%)	856 (47.32%)	<0.01
Age (years)	56.92 (56.51, 57.34)	58.57 (58.16, 58.98)	59.30 (58.88, 59.71)	<0.0001
BMI (kg/m^2^)	22.62 (22.44, 22.79)	23.76 (23.59, 23.94)	24.43 (24.25, 24.60)	<0.0001
Education level, *n* (%)				
No formal education	395 (21.94%)	421 (23.26%)	395 (21.84%)	0.69
Elementary or below	661 (36.72%)	668 (36.91%)	682 (37.70%)	0.52
Middle school or above	744 (41.33%)	721 (39.83%)	732 (40.46%)	0.77
Smoking status, *n* (%)				
Nonsmoker	1187 (65.94%)	1120 (61.88%)	1083 (59.87%)	<0.001
Exsmoker	112 (6.22%)	139 (7.68%)	188 (10.39%)	<0.0001
Current smoker	501 (27.83%)	551 (30.44%)	538 (29.74%)	0.40
Drinking status, *n* (%)				
≥1 time/month	460 (25.56%)	423 (23.37%)	426 (23.55%)	0.28
<1 time/month	151 (8.39%)	155 (8.56%)	132 (7.30%)	0.16
Nondrinker	1189 (66.06%)	1232 (68.07%)	1251 (69.15%)	0.07
Fasting plasma glucose, *n* (%)				
<110 mg/dL	1399 (77.72%)	1298 (71.71%)	1227 (67.83%)	<0.0001
110-126 mg/dL	263 (14.61%)	290 (16.02%)	316 (17.47%)	0.02
≥126 mg/dL	138 (7.67%)	222 (12.27%)	266 (14.70%)	<0.0001
Income ≥2433.07 yuan/year, *n* (%)	314 (17.44%)	261 (14.42%)	261 (14.43%)	0.05
Married, *n* (%)	1621 (90.06%)	1612 (89.06%)	1592 (88.00%)	0.06
Urban, *n* (%)	534 (29.67%)	613 (33.87%)	670 (37.04%)	<0.0001
Hypertension	398 (22.11%)	507 (28.01%)	615 (34.00%)	<0.0001
Total cholesterol (mg/dL)	190.15 (188.42, 191.87)	194.82 (193.10, 196.54)	196.31 (194.59, 198.03)	<0.0001
Triglycerides (mg/dL)	112.22 (107.62, 116.83)	133.98 (129.39, 138.57)	140.90 (136.31, 145.49)	<0.0001
HDL cholesterol (mg/dL)	55.14 (54.45, 55.83)	50.64 (49.95, 51.33)	48.66 (47.97, 49.35)	<0.0001
eGFR (mL/min per 1.73 m^2^)	81.71 (81.27, 82.14)	80.04 (79.61, 80.47)	79.68 (79.24, 80.11)	<0.0001
Baseline uric acid (mg/dL)	3.99 (3.94, 4.04)	4.25 (4.20, 4.29)	4.42 (4.37, 4.47)	<0.0001

^a^CRP: C-reactive protein; BMI: body mass index; HDL: high-density lipoprotein; eGFR: estimate glomerular filtration rate. ^b^Analysis of variance or logistic regression analysis. ^c^Least square mean (95% confidence interval) (all such values).

**Table 2:** Association between high-sensitivity CRP (mg/L) and hyperuricemia^a^.

	Tertiles of high-sensitivity CRP (mg/L)			*P* trend^b^
	Tertile 1	Tertile 2	Tertile 3	
No. of hyperuricemia	91	166	217	
No. of participants	1800	1810	1809	
Crude model	Reference	1.90 (1.46, 2.48)^c^	2.56 (1.99, 3.31)	<0.0001
Adjusted model 1^d^	Reference	1.67 (1.28, 2.19)	2.09 (1.61, 2.72)	<0.0001
Adjusted model 2^e^	Reference	1.25 (0.94, 1.68)	1.36 (1.02, 1.82)	0.08

^a^CRP: C-reactive protein; BMI: body mass index; HDL: high-density lipoprotein; eGFR: estimate glomerular filtration rate. ^b^Analysis of variance or logistic regression analysis. ^c^Odds ratio (95% confidence interval) (all such values). ^d^Adjusted for age, sex, BMI. ^e^Additionally adjusted for education level, smoking status, drinking status, level of income, marital status, fasting blood glucose levels, living area, baseline uric acid, hypertension, total cholesterol, triglycerides, HDL cholesterol, and eGFR on model 1.

**Table 3:** Association between high-sensitivity CRP (mg/L) and change in uric acid concentrations (mg/dL)^a^.

	Tertiles of high-sensitivity CRP (mg/L)			*β*	Standard error	*t* value	*P* ^b^
	Tertile 1 (*n* = 1800)	Tertile 2 (*n* = 1810)	Tertile 3 (*n* = 1809)				
Crude model	0.56 (0.51, 0.60)^c^	0.59 (0.55, 0.64)	0.63 (0.58, 0.67)	0.035	0.017	2.09	0.04
Adjusted model 1^d^	0.58 (0.53, 0.62)	0.59 (0.54, 0.64)	0.61 (0.56, 0.65)	0.015	0.017	0.90	0.37
Adjusted model 2^e^	0.55 (0.50, 0.59)	0.59 (0.55, 0.64)	0.63 (0.59, 0.68)	0.044	0.017	2.61	<0.01

^a^CRP: C-reactive protein; BMI: body mass index; HDL: high-density lipoprotein; eGFR: estimate glomerular filtration rate. ^b^Analysis of multiple linear regression. ^c^Least square mean (95% confidence interval) (all such values); analysis of covariance; change in uric acid concentrations (mg/dL) was calculated using data in 2015 minus data in 2011. ^d^Adjusted for age, sex, BMI. ^e^Additionally adjusted for education level, smoking status, drinking status, level of income, marital status, fasting blood glucose levels, living area, baseline uric acid, hypertension, total cholesterol, triglycerides, HDL cholesterol, and eGFR based on model 1.

**Table 4:** Stratified analyses for association between high-sensitivity CRP (mg/L) and hyperuricemia^a^.

	Tertiles of high-sensitivity CRP (mg/L)			*P* trend^b^	*P* for interaction^b^
	Tertile 1	Tertile 2	Tertile 3		
Sex					0.13
Male (*n* = 2406)					
No. of hyperuricemia	58	91	98		
No. of participants	796	808	802		
High-sensitivity CRP (mg/L)	0.44 (0.43, 0.44)^c^	1.00 (0.99, 1.02)	3.29 (3.16, 3.42)		
Adjusted model 2^d^	Reference	1.24 (0.85, 1.83)^e^	1.19 (0.81, 1.76)	0.59	
Female (*n* = 3013)					
No. of hyperuricemia	33	76	118		
No. of participants	1004	1003	1006		
High-sensitivity CRP (mg/L)	0.41 (0.40, 0.42)	0.92 (0.91, 0.94)	3.01 (2.90, 3.12)		
Adjusted model 2	Reference	1.45 (0.93, 2.30)	1.69 (1.10, 2.66)	0.04	
Age					0.37
Age ≥60 years (*n* = 2293)					
No. of hyperuricemia	44	76	98		
No. of participants	762	763	768		
High-sensitivity CRP (mg/L)	0.46 (0.45, 0.47)	1.06 (1.04, 1.07)	3.43 (3.30, 3.57)		
Adjusted model 2	Reference	1.24 (0.81, 1.92)	1.45 (0.96, 2.21)	0.10	
Age <60 years (*n* = 3126)					
No. of hyperuricemia	53	85	118		
No. of participants	1040	1029	1057		
High-sensitivity CRP (mg/L)	0.40 (0.39, 0.41)	0.88 (0.87, 0.89)	2.88 (2.78, 2.98)		
Adjusted model 2	Reference	1.16 (0.79, 1.71)	1.21 (0.83, 1.78)	0.43	
BMI					0.40
Normal BMI (*n* = 3178)					
No. of hyperuricemia	45	78	99		
No. of participants	1056	1060	1062		
High-sensitivity CRP (mg/L)	0.37 (0.37, 0.38)	0.81 (0.80, 0.82)	2.91 (2.79, 3.02)		
Adjusted model 2	Reference	1.14 (0.76, 1.75)	1.18 (0.78, 1.79)	0.56	
High BMI (*n* = 1605)					
No. of hyperuricemia	33	60	67		
No. of participants	532	538	535		
High-sensitivity CRP (mg/L)	0.48 (0.47, 0.49)	1.08 (1.06, 1.10)	3.07 (2.94, 3.21)		
Adjusted model 2	Reference	1.65 (1.02, 2.70)	1.53 (0.96, 2.49)	0.25	
Obesity (*n* = 636)					
No. of hyperuricemia	24	28	40		
No. of participants	211	213	212		
High-sensitivity CRP (mg/L)	0.66 (0.63, 0.68)	1.53 (1.48, 1.57)	4.08 (3.85, 4.32)		
Adjusted model 2	Reference	0.83 (0.43, 1.62)	1.07 (0.57, 2.03)	0.63	

^a^CRP: C-reactive protein; BMI: body mass index; HDL: high-density lipoprotein; eGFR: estimate glomerular filtration rate; OR: odds ratio; CI: confidence interval. ^b^Analysis logistic regression analysis. ^c^Least square mean (95% confidence interval) (all such values). ^d^Adjusted for age, sex, BMI, education level, smoking status, drinking status, level of income, marital status, fasting blood glucose levels, living area, baseline uric acid, hypertension, total cholesterol, triglycerides, HDL cholesterol, and eGFR. ^e^Odds ratio (95% confidence interval) (all such values).

## Data Availability

The data that support the findings of this study are openly available in China Health and Retirement Longitudinal Study at http://charls.pku.edu.cn/index.html.

## Supplementary Materials

Supplementary Materials Table S1: general characteristics of participants according to tertiles of high-sensitivity CRP by age. Table S2: general characteristics of participants according to tertiles of high-sensitivity CRP by sex. Table S3: general characteristics of participants according to tertiles of high-sensitivity CRP by BMI. Figure S1: receiver operating characteristic (ROC) curve to test the predicting ability of the final model. Area under ROC curve was 0.6018.
