# Supplementary material for: Higher Levels of High-Sensitivity C-Reactive Protein Is Positively Associated with the Incidence of Hyperuricemia in Chinese Population: A Report from the China Health and Retirement Longitudinal Study
Source: Mediators Inflamm. 2020 May 20;2020:3854982. doi: 10.1155/2020/3854982 (PMC7256734; doi:10.1155/2020/3854982)
Supplement: Supplementary Materials — Table S1: general characteristics of participants according to tertiles of high-sensitivity CRP by age. Table S2: general characteristics of participants according to tertiles of high-sensitivity CRP by sex. Table S3: general characteristics of participants according to tertiles of high-sensitivity CRP by BMI. Figure S1: receiver operating characteristic (ROC) curve to test the predicting ability of the final model. Area under ROC curve was 0.6018. [file 3854982.f1.docx]

| Table S1 General characteristics of participants according to tertiles of high-sensitivity CRP by age ^a^ | | | |
| --- | --- | --- | --- |
|  | Age ≥ 60 years | Age ＜ 60 years | *P* trend ^b^ |
| Male, n (%) | 1149 (50.11%) | 1257 (40.21%) | < 0.0001 |
| BMI (kg/m²) | 22.94 (22.79, 23.10) ^c^ | 24.09 (23.96, 24.22) | < 0.0001 |
| Education level, n (%) |  |  |  |
| No formal education | 713 (31.09%) | 498 (15.93%) | < 0.0001 |
| Elementary or below | 823 (35.89%) | 1188 (38.00%) | 0.11 |
| Middle school or above | 757 (33.01%) | 1440 (46.07%) | < 0.0001 |
| Smoking status, n (%) |  |  |  |
| Non-smoker | 1311 (57.17%) | 2079 (66.51%) | < 0.0001 |
| Ex-smoker | 248 (10.82%) | 191 (6.11%) | < 0.0001 |
| Current smoker | 734 (32.01%) | 856 (27.38%) | < 0.001 |
| Drinking status, n (%) |  |  |  |
| ≥ 1 time/month | 580 (25.29%) | 729 (23.32%) | 0.09 |
| < 1 time/month | 147 (6.41%) | 291 (9.31%) | < 0.001 |
| Non-drinker | 1566 (68.29%) | 2106 (67.37%) | 0.47 |
| Fasting plasma glucose, n (%) |  |  |  |
| < 110 mg/dL | 1599 (69.73%) | 2325 (74.38%) | < 0.001 |
| 110 - 126 mg/dL | 404 (17.62%) | 465 (14.88%) | < 0.01 |
| ≥ 126 mg/dL | 290 (12.65%) | 336 (10.75%) | 0.03 |
| Income ≥ 2433.07 yuan/year, n (%) | 143 (6.24%) | 693 (22.17%) | < 0.0001 |
| Married, n (%) | 1878 (81.90%) | 2947 (94.27%) | < 0.0001 |
| Urban, n (%) | 722 (31.49%) | 1095 (35.03%) | < 0.01 |
| Hypertension | 811 (35.37%) | 709 (22.68%) | < 0.0001 |
| Total cholesterol (mg/dL) | 194.89 (193.36, 196.43) | 192.94 (191.63, 194.25) | 0.06 |
| Triglycerides (mg/dL) | 121.35 (117.25, 125.45) | 134.72 (131.21, 138.23) | < 0.0001 |
| HDL cholesterol (mg/dL) | 52.65 (52.03, 53.27) | 50.61 (50.08, 51.14) | < 0.0001 |
| eGFR (mL/min per 1.73 m^2^) | 76.71 (76.35, 77.07) | 83.23 (82.92, 83.54) | < 0.0001 |
| Uric acid (mg/dL) | 4.33 (4.29, 4.38) | 4.14 (4.10, 4.17) | < 0.0001 |

^a^ CRP, C-reactive protein; BMI, body mass index; HDL, high-density lipoprotein; eGFR, estimate glomerular filtration rate.

^b^ Analysis of variance or logistic regression analysis.

^c^ Least square mean (95% confidence interval) (all such values).

| Table S2 General characteristics of participants according to tertiles of high-sensitivity CRP by sex ^a^ | | | |
| --- | --- | --- | --- |
|  | Male | Female | *P* trend ^b^ |
| Age (years) | 59.57 (59.21, 59.92) ^c^ | 57.23 (56.91, 57.55) | < 0.0001 |
| BMI (kg/m²) | 23.00 (22.85, 23.15) | 24.09 (23.95, 24.22) | < 0.0001 |
| Education level, n (%) |  |  |  |
| No formal education | 915 (38.03%) | 296 (9.82%) | < 0.0001 |
| Elementary or below | 800 (33.25%) | 1211 (40.19%) | < 0.0001 |
| Middle school or above | 691 (28.72%) | 1506 (49.98%) | < 0.0001 |
| Smoking status, n (%) |  |  |  |
| Non-smoker | 586 (24.36%) | 2804 (93.06%) | < 0.0001 |
| Ex-smoker | 390 (16.21%) | 49 (1.63%) | < 0.0001 |
| Current smoker | 1430 (59.43%) | 160 (5.31%) | < 0.0001 |
| Drinking status, n (%) |  |  |  |
| ≥ 1 time/month | 1092 (45.39%) | 217 (7.20%) | < 0.0001 |
| < 1 time/month | 288 (11.97%) | 150 (4.98%) | < 0.0001 |
| Non-drinker | 1026 (42.64%) | 2646 (87.82%) | < 0.0001 |
| Fasting plasma glucose, n (%) |  |  |  |
| < 110 mg/dL | 1712 (71.16%) | 2212 (73.42%) | 0.06 |
| 110 - 126 mg/dL | 406 (16.87%) | 463 (15.37%) | 0.13 |
| ≥ 126 mg/dL | 288 (11.97%) | 338 (11.22%) | 0.39 |
| Income ≥ 2433.07 yuan/year, n (%) | 545 (22.65%) | 291 (9.66%) | < 0.0001 |
| Married, n (%) | 2186 (90.86%) | 2639 (87.59%) | < 0.001 |
| Urban, n (%) | 774 (32.17%) | 1043 (34.62%) | 0.06 |
| Hypertension | 673 (27.97%) | 847 (28.11%) | 0.91 |
| Total cholesterol (mg/dL) | 188.12 (186.64, 189.60) | 198.27 (196.95, 199.60) | < 0.0001 |
| Triglycerides (mg/dL) | 122.29 (118.28, 126.29) | 134.47 (130.90, 138.05) | < 0.0001 |
| HDL cholesterol (mg/dL) | 50.97 (50.37, 51.58) | 51.87 (51.33, 52.42) | 0.03 |
| eGFR (mL/min per 1.73 m^2^) | 81.32 (80.95, 81.70) | 79.79 (79.46, 80.13) | < 0.0001 |
| Uric acid (mg/dL) | 4.71 (4.67, 4.75) | 3.83 (3.80, 3.86) | < 0.0001 |

^a^ CRP, C-reactive protein; BMI, body mass index; HDL, high-density lipoprotein; eGFR, estimate glomerular filtration rate.

^b^ Analysis of variance or logistic regression analysis.

^c^ Least square mean (95% confidence interval) (all such values).

| Table S3 General characteristics of participants according to tertiles of high-sensitivity CRP by BMI ^a^ | | | | |
| --- | --- | --- | --- | --- |
|  | BMI ＜ 24 | 24 ≤ BMI ＜ 28 | BMI ≥ 28 | *P* trend ^b^ |
| Male, n (%) | 1586 (49.91%) | 631 (39.31%) | 189 (29.72%) | < 0.0001 |
| Age (years) | 59.39 (59.08, 59.70) ^c^ | 56.95 (56.51, 57.38) | 55.97 (55.28, 56.66) | < 0.0001 |
| Education level, n (%) |  |  |  |  |
| No formal education | 834 (26.24%) | 287 (17.88%) | 90 (14.15%) | < 0.0001 |
| Elementary or below | 1172 (36.88%) | 605 (37.69%) | 234 (36.79%) | 0.83 |
| Middle school or above | 1172 (36.88%) | 713 (44.42%) | 312 (49.06%) | < 0.0001 |
| Smoking status, n (%) |  |  |  |  |
| Non-smoker | 1820 (57.27%) | 1089 (67.85%) | 481 (75.63%) | < 0.0001 |
| Ex-smoker | 240 (7.55%) | 145 (9.03%) | 54 (8.49%) | 0.15 |
| Current smoker | 1118 (35.18%) | 371 (23.12%) | 101 (15.88%) | < 0.0001 |
| Drinking status, n (%) |  |  |  |  |
| ≥ 1 time/month | 863 (27.16%) | 351 (21.87%) | 95 (14.94%) | < 0.0001 |
| < 1 time/month | 262 (8.24%) | 128 (7.98%) | 48 (7.55%) | 0.54 |
| Non-drinker | 2053 (64.60%) | 1126 (70.16%) | 493 (77.52%) | < 0.0001 |
| Fasting plasma glucose, n (%) |  |  |  |  |
| < 110 mg/dL | 2441 (76.81%) | 1095 (68.22%) | 388 (61.01%) | < 0.0001 |
| 110 - 126 mg/dL | 456 (14.35%) | 282 (17.57%) | 131 (20.60%) | < 0.0001 |
| ≥ 126 mg/dL | 281 (8.84%) | 228 (14.21%) | 117 (18.40%) | < 0.0001 |
| Income ≥ 2433.07 yuan/year, n (%) | 465 (14.63%) | 287 (17.88%) | 84 (13.21%) | 0.55 |
| Married, n (%) | 2762 (86.91%) | 1478 (92.09%) | 585 (91.98%) | < 0.0001 |
| Urban, n (%) | 921 (28.98%) | 625 (38.94%) | 271 (42.61%) | < 0.0001 |
| Hypertension | 726 (22.84%) | 529 (32.96%) | 265 (41.67%) | < 0.0001 |
| Total cholesterol (mg/dL) | 191.20 (189.90, 192.50) | 197.07 (195.25, 198.90) | 198.24 (195.35, 201.14) | < 0.0001 |
| Triglycerides (mg/dL) | 112.35 (108.94, 115.76) | 144.33 (139.54, 149.13) | 174.06 (166.44, 181.68) | < 0.0001 |
| HDL cholesterol (mg/dL) | 54.99 (54.48, 55.50) | 47.57 (46.86, 48.28) | 43.75 (42.62, 44.88) | < 0.0001 |
| eGFR (mL/min per 1.73 m^2^) | 80.59 (80.26, 80.92) | 80.31 (79.85, 80.77) | 80.31 (79.57, 81.04) | 0.56 |
| Uric acid (mg/dL) | 4.17 (4.13, 4.20) | 4.27 (4.22, 4.32) | 4.36 (4.28, 4.44) | < 0.0001 |

^a^ CRP, C-reactive protein; BMI, body mass index; HDL, high-density lipoprotein; eGFR, estimate glomerular filtration rate.

^b^ Analysis of variance or logistic regression analysis.

^c^ Least square mean (95% confidence interval) (all such values).


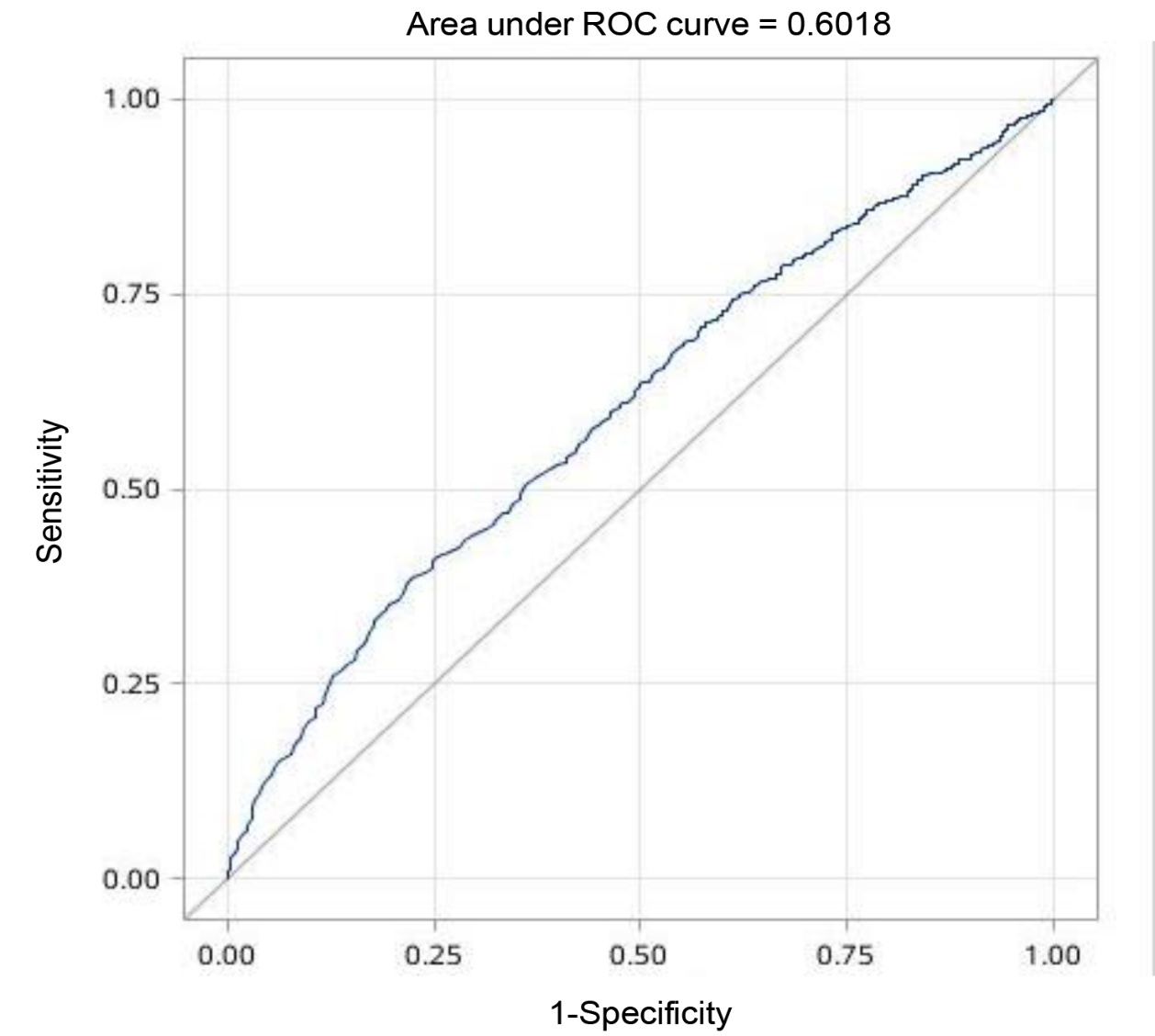


Figure S1 Receiver operating characteristic (ROC) curve to test the predicting ability of the final model. Area under ROC curve was 0.6018.
